# Measurement of Δ^9^THC and metabolites in the brain and peripheral tissues after intranasal instillation of a nanoformulation

**DOI:** 10.1186/s42238-022-00171-8

**Published:** 2023-02-07

**Authors:** Gunjan Upadhyay, Oksana Fihurka, Connor Habecker, Pranav Patel, Juan Sanchez-Ramos

**Affiliations:** 1 SGN Nanopharma, Inc, 3720 Spectrum Blvd, Tampa, FL USA; 2 Department of Neurology, University of South Florida, Tampa, FL USA

**Keywords:** Intranasal administration, Nanoparticles, Nanoemulsions, Delta-9-tetrahydrocannabinol (Δ^9^THC), Cannabidiol (CBD), Pharmacokinetics

## Abstract

**Background:**

Comparative bioavailability of cannabinoids following their administration by dosing routes has been studied previously, but there are no quantitative reports of distribution of Δ^**9**^THC, nor its metabolites, across various brain regions following intranasal (i.n.) administration. The aim of the present study was to determine the time course of Δ^**9**^THC transport from nose to brain and to quantify the distribution of Δ^**9**^THC and its metabolites in four brain regions.

**Methods:**

Δ^9^THC was formulated as a lipophilic nano-emulsion and instilled i.n. to three groups of adult mice and euthanized after 2, 4, and 8 h. Brains were dissected into 4 regions. Sensitive analytical methods (HPLC-MS) were utilized to quantify levels of Δ^**9**^THC and metabolites in brain regions and peripheral tissues. Data was expressed as mean concentrations (± SEM) of Δ^**9**^THC and metabolites in brain regions, blood, plasma, urine, and liver. Two-way analysis of variance was performed followed by post hoc multiple comparisons.

**Results:**

Peak concentrations of Δ^**9**^THC were reached at 2 h in the brain (15.9 ng/mg), blood (4.54 μg/mL), and plasma (4.56 μg /mL). The percentage of administered dose of Δ^**9**^THC transported to the brain (5.9%) was greater than in blood (1.7%), plasma (1.6%), urine (0.4%), and liver (0.1%). Concentrations of Δ^9^THC and its THC-COOH metabolite in the liver reached their highest levels at 8 h.

**Discussion:**

The present study is the first to report the uptake and distribution across brain regions of Δ^**9**^THC and its principal metabolites following i.n. administration. The systemic bioavailability (absorption into the blood) of intranasal Δ^**9**^THC was 1.7% of the administered dose, much lower than that reported by others after oral ingestion (7–10%) and inhalation (20–35%), but those prior studies did not measure the transport of Δ^**9**^THC into brain regions. Others have reported Δ^**9**^THC in the whole brain following i.n. instillation in a different species (rats) to be twice (5.9%) that following i.p. injections, while metabolites of Δ^**9**^THC in rat brain were lower after i.n. administration.

**Conclusions:**

The intranasal route of a Δ^**9**^THC nanoformulation is an effective way to deliver cannabinoids to the brain, especially in those who cannot take the medication orally. Going forward, a metered dosing nasal spray will provide accurate and consistent doses.

**Supplementary Information:**

The online version contains supplementary material available at 10.1186/s42238-022-00171-8.

## Background

Cannabis in various formulations can be administered by many routes, including inhalation, oral ingestion, sub-lingual, intranasal (i.n.) insufflation, topical application to the skin, and by rectal suppository (Baglot et al., 2021; Grotenhermen, 2003; Mahmoudinoodezh et al., 2022; Polidoro et al., 2022). A recent review article summarized the disposition and extent of penetration of the cannabinoids Δ^**9**^THC and CBD into the brain in animal models after various routes of administration (Calapai et al., 2020). However, little research has been conducted on i.n. administration which provides an alternative route for those with nausea or vomiting and who need a more rapid onset of action that follows the oral route.

Human studies focus on the bioavailability of Δ^**9**^THC (absorption into the systemic circulation) following smoking or ingesting cannabis. Determination of penetration into the brain in humans cannot be carried out in living subjects. Δ^**9**^THC is rapidly absorbed into the circulation after smoking with a bioavailability of 18–50%; peak levels in the plasma were attained within a few minutes (Lindgren et al., 1981). In another report, inhalation of smoke from a cigarette with about 16–34 mg of Δ^**9**^THC produced peaks with mean concentrations ranging between 84.3 and 162.2 μg/L. Then, concentrations readily decreased down to 1–4 μg/L after 3 to 4 h (Huestis et al., 1992). The rapid onset of action of inhaled cannabis may make it appropriate for treatment of some forms of acute pain. One study tested the effects of smoked cannabis (low, medium, or high doses vs. inactive placebo) on intradermal capsaicin-induced pain responses using a randomized, double-blind, crossover trial in 15 healthy volunteers (Wallace et al., 2007). Results indicated a significant decrease in pain with the medium cannabis dose and a significant increase in pain with the high dose. The authors concluded that there is likely a therapeutic window of modest analgesia for smoked cannabis.

After oral administration to humans, Δ^**9**^THC was reported to be absorbed into the systemic circulation more slowly and unpredictably with a peak in concentration generally obtained after one to 3 h (Law et al., 1984). In another study, oral ingestion resulted in THC peaks in plasma of 0.58–12.48 μg/L, 2.7–6.3 μg/L, and 4.4–11 μg/L, after THC intake of 2.5, 15, and 20 mg, respectively (Grotenhermen, 2003). The longer duration of action following oral routes makes this approach preferable for the management of chronic pain syndromes and arthritis (Hill et al., 2017).

Intramuscular administration of 30 mg Δ^**9**^THC in rats resulted in a very low bioavailability (0.06%) (Nahas, 2001). Despite a bioavailability of inhaled and oral Δ^**9**^THC of 20% and 6%, respectively, the quantity delivered to the brain is less than 1% of these percentages. Yet, the small amount that penetrates brain is psychoactive, indicating the high chemical potency related to the bioactivity of Δ^**9**^THC in the central nervous system (Cabral & Jamerson, 2014).

The i.n. route has been studied much less frequently, but has several advantages over the oral route (Ahmed et al., 2022). The nasal mucosa is thin and highly vascularized, allowing the drug to penetrate the single epithelial cell layer directly into the systemic blood circulation avoiding first-pass hepatic and intestinal metabolism. The end result is a more rapid access to the brain and onset of therapeutic effects compared to the oral route. In addition, intranasal instillation of cannabinoids may be more acceptable for patients who experience nausea, vomiting, oral mucositis, and impaired gastrointestinal function. However, until the present report, no other investigators have measured the transport of Δ^**9**^THC from the nose to the brain and quantified the distribution of Δ^**9**^THC and its metabolites across brain regions over time.

The focus of this report centers on testing a nanoformulation of Δ^**9**^THC for the capacity to transport the agent from the nose to the brain and to determine regional brain concentrations of Δ^**9**^THC and metabolites over time. We expect that the intranasal route will be useful for those who patients who are nauseous or vomiting, and for those who need a more rapid onset of therapeutic effects than when given orally.

## Materials and methods

### Preparation of nanoformulation of Δ^9^THC

Δ^9^THC (3.2537 g) was added to a beaker. To this 0.625 g of 100% Ethanol (Lot no-007116 , Decon Lab, King of Prussia, PA) was added and sonicated until Δ^9^THC was dissolved. This was followed by the addition of 7.5 g of GTCC MBAL SG oil (Lot no- 42733, Crodamol, Plainsboro, NJ) and 2.7 g of polysorbate 80 (Lot no-1186606, Croda, Plainsboro, NJ). The mixture was sonicated until dissolved. This is the oil phase. In another beaker, 0.06 g of ascorbic acid (Lot no-181977 Fisher Chemicals, Waltham, MA) was dissolved in 18 g of Milli-Q water. This water was added to the oil phase while stirring using an electric stirrer for about 15 minutes to make it an emulsion. After this, the pH of this solution was set around 7 using a diluted sodium hydroxide solution. This emulsion was passed through the homogenizer (Make-Avestin, Emulsiflex C3) for 3 cycles at 10,000–15,000 psi. The particle size of this formulation was found to be an average of 150 nm. The concentration Δ^9^THC in the nanoformulation was 8% by weight. The cannabinoid metabolites 11-hydroxy-THC (THC-OH) and tetrahydrocannabinol carboxylic acid (Δ^9^THC-COOH) were purchased from Cayman Chemicals (Ann Arbor, MI).

### Intranasal administration

All procedures with animals were performed in accordance with the Institutional Animal Care and Use Committee (IACUC) approved protocol. Adult male mice from the FBV/N strain (3-6 mos) were purchased from Jackson Laboratories (Bar Harbor, ME, USA). The mice were housed under standard conditions with free access to water and food. Mice were lightly anesthetized with isoflurane prior to performing intranasal instillation. The animals were held by the scruff with the nose positioned to facilitate i.n. dosing. The Δ^9^THC nanoformulation (in 6μl aliquots ) was instilled drop by drop into one nostril and after 5 s, the same step was repeated for the other nostril, then the mouse was placed back to the anesthesia cabinet.

Each animal received a total of 126 μL of nanoformulation containing 10.22 mg Δ^9^THC. The administered per mouse = 10.22 mg per 38 g mouse = 0.268 mg/g = 268 μg/g. Following administration, three groups of mice (total *n*= 22) were sacrificed at 2 h (*n*=8), 4 h (*n*=8), and 8 h (*n*=6) after treatment. Liver, lungs, brain, blood, and urine were collected for analysis. Tubes were kept on ice and centrifuged at 300 × g for 5 minutes; serum samples were then immediately separated. Tissue sampling of the olfactory bulb, cerebral cortex, corpus striatum, and hippocampus was performed by the brain dissection. All samples were kept frozen at −80 °C until assayed.

### Sample preparation

Olfactory bulb (OB), hippocampus (HP), striatum (ST), cortex (CX), and liver were weighed individually, and an appropriate amount of organic solvent was added. Acetonitrile was added to the blood, plasma, and urine sample, vortexed for about 5 min, and kept at −30°C for 15 min to precipitate the proteins. All The samples were centrifuged at 4500 rpm for 15 min and the supernatant was injected into the LC-MS.

### Chromatographic conditions

Manufacturers suggested methods for chromatography were followed.

(Ref: https://www.waters.com/webassets/cms/library/docs/720005769en.pdf)

**Fig. 1:**
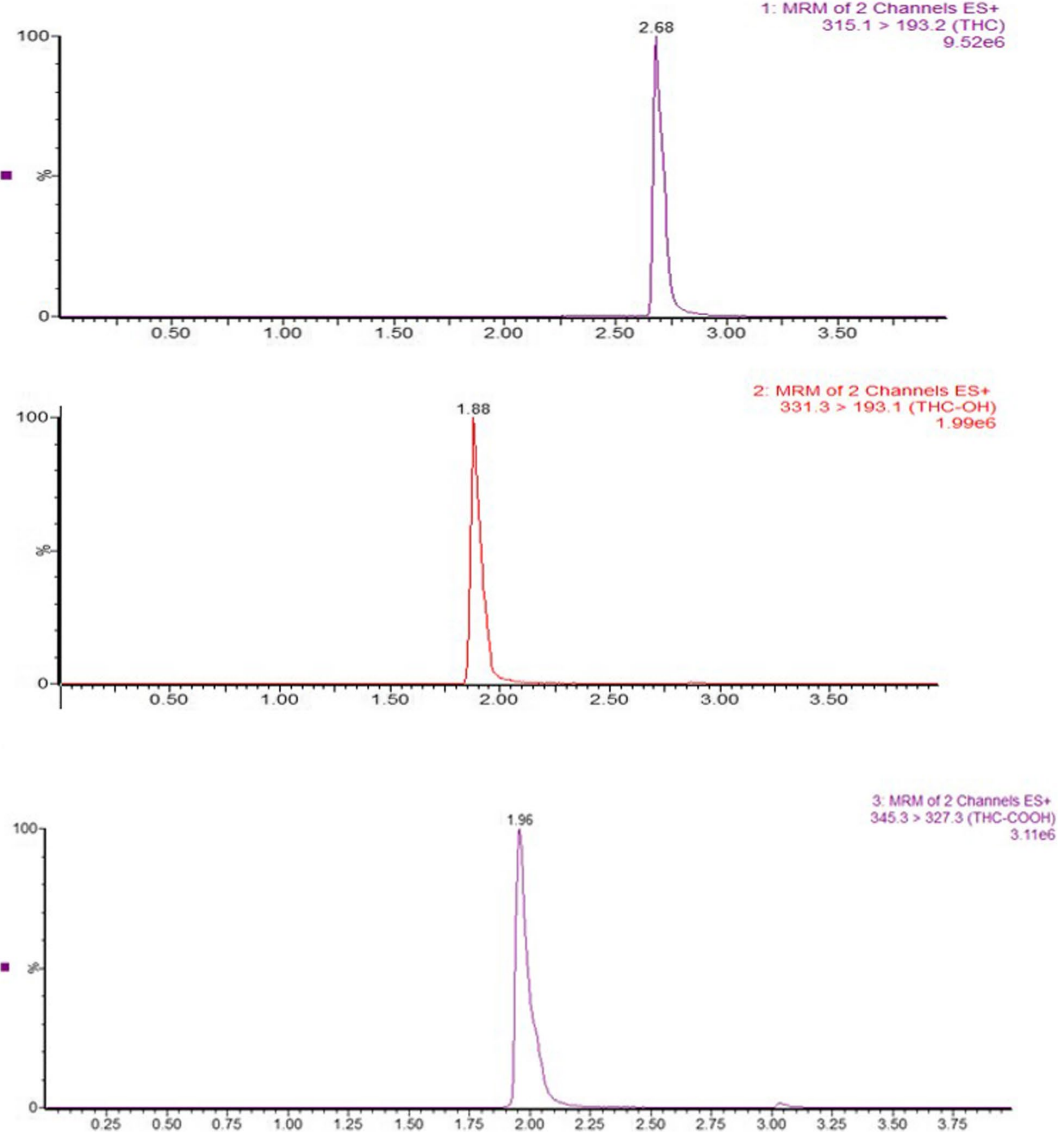
Retention time of Δ^9^THC, THC-OH, and THC-COOH

**Fig. 2:**
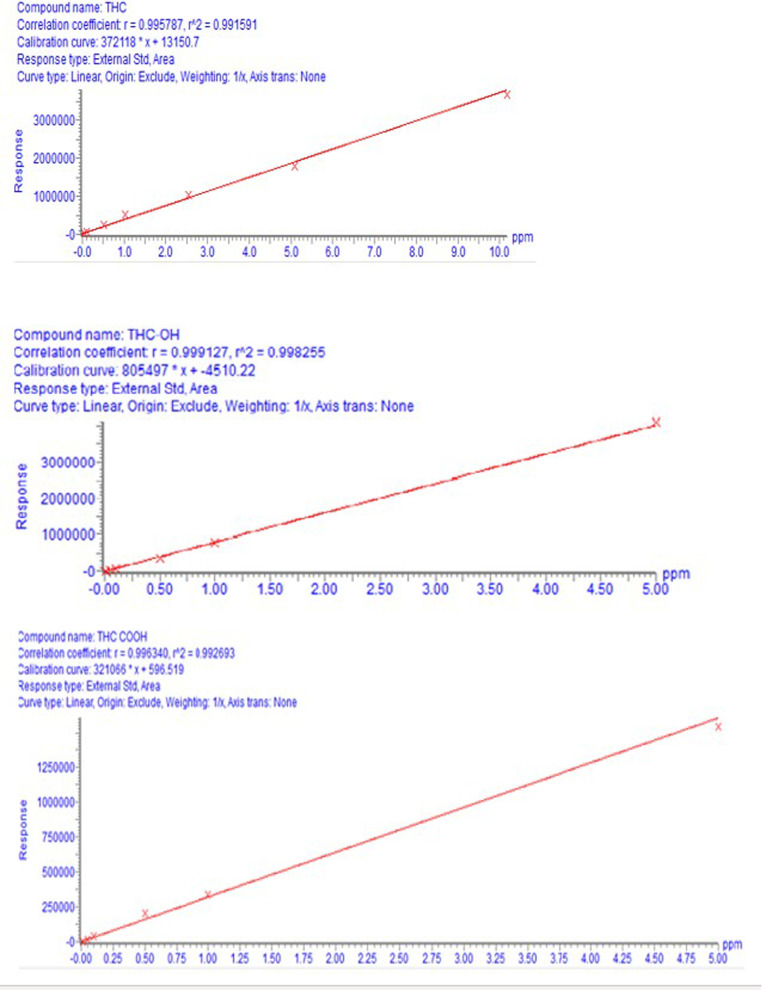
Standard calibration curve for Δ^9^THC, THC-OH, and THC-COOH

Samples (3μL) were injected in the Waters UPLC Class-I using 0.1% formic acid in water (A) and 0.1% formic acid in acetonitrile (B), equipped with the acquity BEH C-18 column (50 mm × 2.1 × 1.7 μm) at the temperature of 40°C. The initial condition for the gradient was set to 50% B for 1 min and then increased linearly to 95% B till 3 min, kept constant till 3.5 min. The column was set back to initial conditions at 3.6 min and saturated with the mobile phase for next injection for 24 s. The separation of THC and its metabolites was carried out using a flow rate of 0.6 mL/min. The sample temperature was kept at 5°C to prevent degradation of the samples. The run time was 4 min/sample (see Figs. 1 and 2).

To avoid false positive results, the control mice samples were extracted and injected in the LC-MS as blanks and the results were auto-calculated using the Masslynx software to avoid false positive results for each organ. Extracting solvent was run before and after each organ to make sure there was no carryover.

### Mass Spectrophotometer conditions

ESI positive ionization mode at the capillary voltage of 2 k.v was set using Xevo-TQ-S mass spectrophotometer. The desolvation gas and temperature was set to 1000 L/h and 500°C. Cone gas was set to 150 L/h and the source temperature was set to 150°C. The cone voltage was optimized for different analytes.

All the analysis was carried out using Mass Lynx software and blanks and placebos were injected to make sure there is no false positive results. The carryover was also confirmed by using a high concentration of the standard stock solution and the blanks were run after that.

### Statistical analysis

For an overall estimate of extent of transport of the drug to the brain following intranasal administration, the mean concentration of Δ^**9**^THC plus metabolites in the four brain regions was expressed as a percentage of the intranasal dose administered (268 μg/g per mouse). All data are expressed as mean ± SEM. Statistical data analysis and graphing utilized Prism 9 (GraphPad Software, LLC). Concentrations of Δ^**9**^THC and THC metabolites were analyzed by 2-way ANOVA with brain regions as one factor and time (2, 4, and 8 h) as the second factor. Post hoc comparisons used Dunnet’s or Sidak’s multiple comparison tests and differences were considered significant at *p* ≤ 0.05.

## Results

**Fig. 3:**
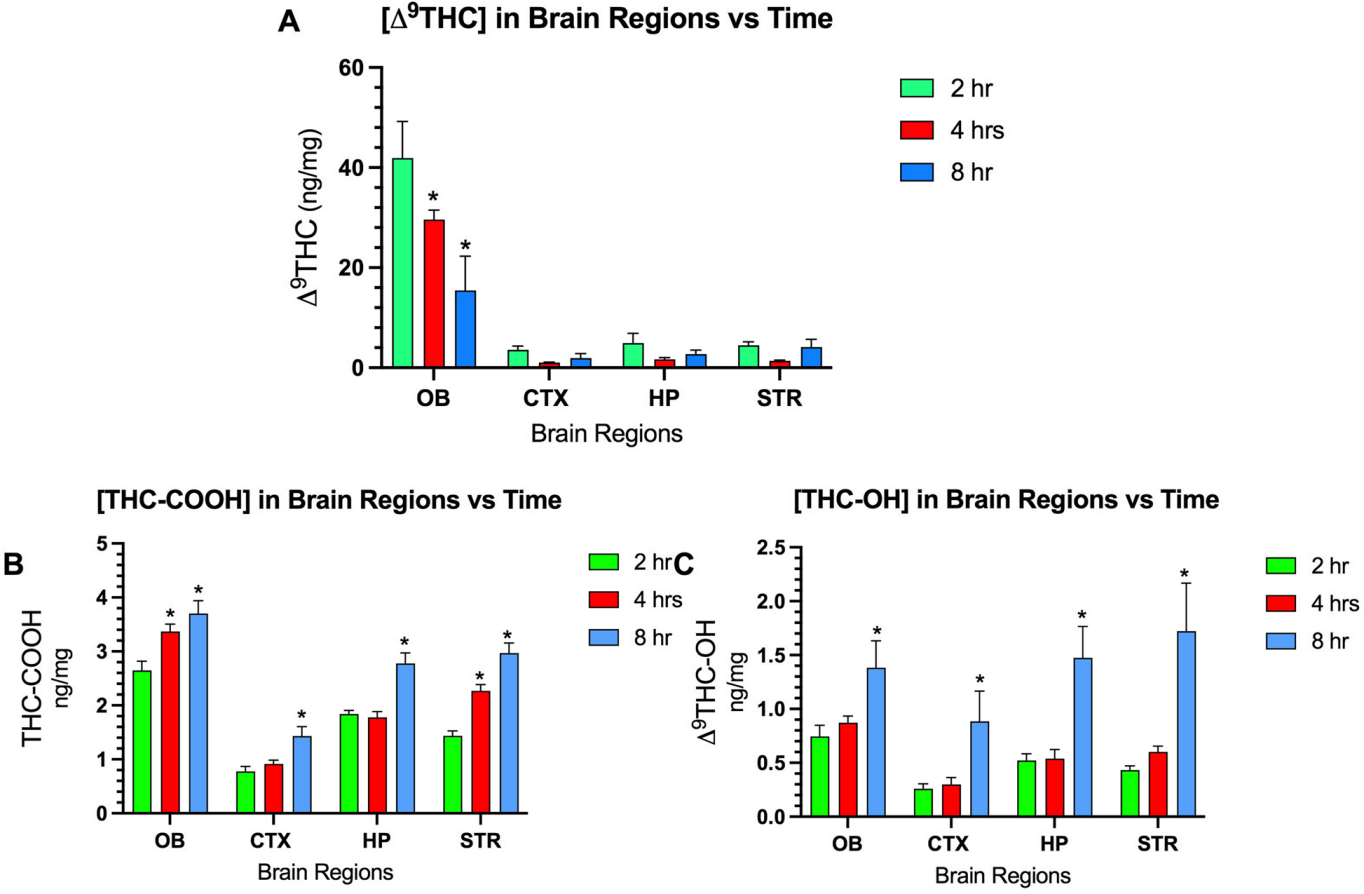
**A** Changes over time of concentrations of Δ^**9**^THC (ng/mg) in four brain regions following intranasal instillation. Abbreviations: olfactory bulb (OB), cerebral cortex (CTX), hippocampus (HP), and striatum (STR). **B** Changes over time of THC-COOH and **C** Δ^**9**^THC-OH. Two-way ANOVA analysis (brain regions vs time) was statistically significant for each set of data Δ^**9**^THC, THC-OH, and THC=COOH (*p*=0.05). Each panel showed that the brain region accounted for the greatest percentage of the total variance in levels of Δ^**9**^THC, THC-COOH, and THC-OH. *N*=8 mice per each cohort euthanized at 2 and 4 h; *N*=6 for the 8-h cohort. Dunnett’s multiple comparison reveals significant differences comparing levels at 4 and 8 h compared to levels at 2 h (* = *p*< 0.05)

The concentrations of Δ^**9**^THC and its metabolites (THC-OH, THC-COOH) across 4 brain regions after i.n. administration is shown in Fig. 3. The highest concentrations of Δ^**9**^THC (45 ng/mg) were measured in the olfactory bulb at 2 h with a gradual decline over time (Fig 3A). Levels in the other 3 brain regions remained below 4 ng/mg at all 3 time points. Concentrations of the metabolites THC-OH and THC-COOH increased in all brain regions over time reaching a peak at 8 h after administration (Fig. 3B and C).

**Fig. 4:**
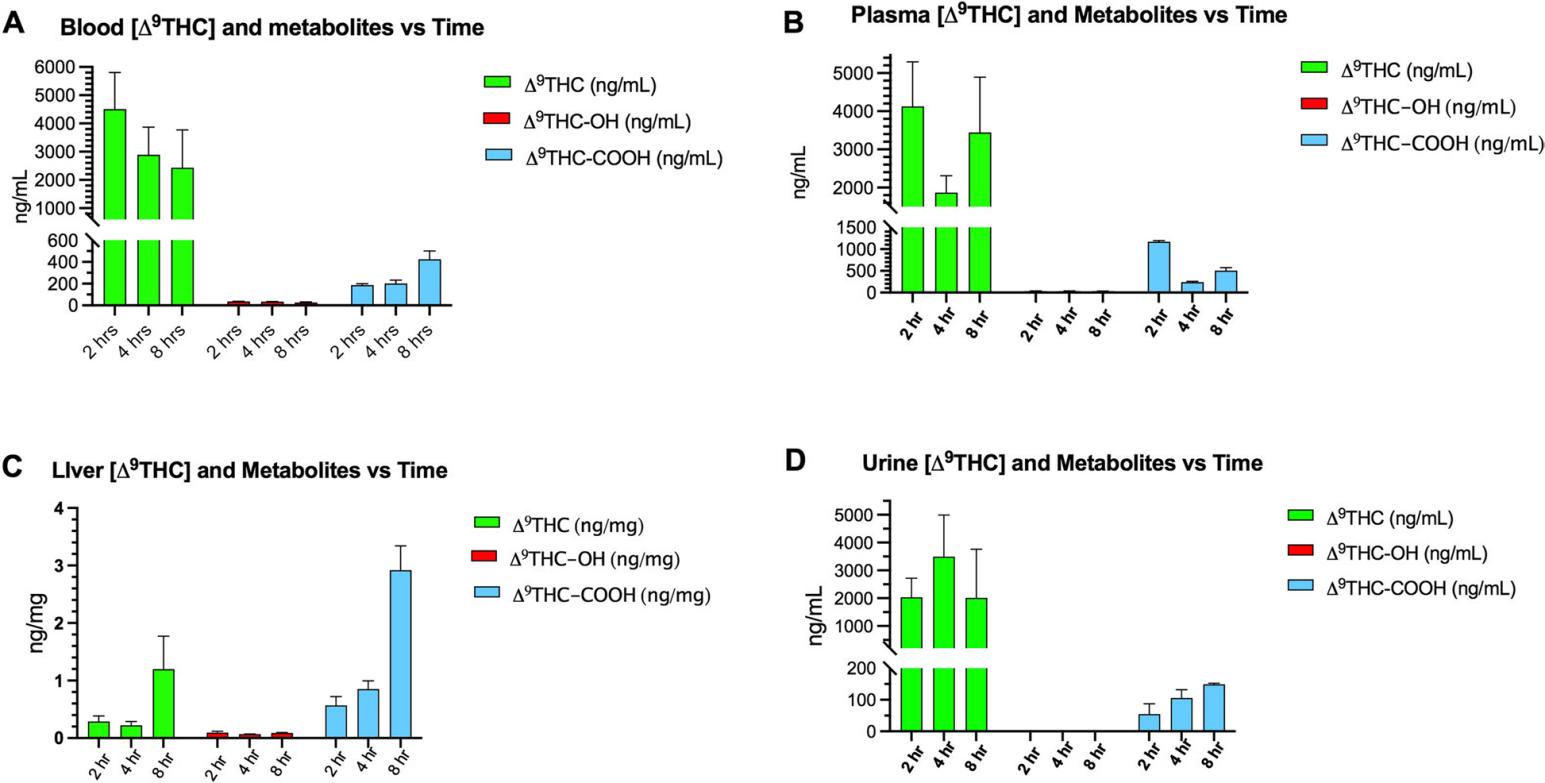
Changes over time of concentrations of Δ^**9**^THC and metabolites in the **A** blood, **B** plasma, **C** liver, and **D** urine. Two-way analysis ANOVA (metabolites vs time) was statistically significant for each panel (*p*=0.05) with metabolites accounting for the greatest percentage of total variance in levels of Δ^**9**^THC, Δ^**9**^THC-COOH, and Δ^**9**^THC-OH

The concentrations of Δ^**9**^THC and metabolites in the blood, plasma, liver, and urine were also measured at 2, 4, and 8 h (Fig. 4). The lowest levels of Δ^**9**^THC were measured in the liver, but that organ accumulated the highest levels of the THC-COOH metabolite at 8 h. THC-OH metabolite in those tissues was extremely low (barely detectable).

Comparison of concentrations of Δ^**9**^THC in the brain and peripheral tissues attained after 2 h is shown in Table 1. Notably, the percentage of the administered dose of Δ^**9**^THC was highest in the brain and lowest in the liver, indicating that the i.n. route of administration is highly effective in delivering the drug to brain tissue.

**Table 1:** Δ^9^THC concentration in various tissues at 2 h following i.n. instillation^a^

	2 h	% of dose administered
Mean of 4 brain regions	15.9 ± 9.8 ng/mg (15.9 μg/g)	5.9%
Blood	4.56 ± 1.29 μg/mL	1.7%
Plasma	4.54 ± 1.36 μg/mL	1.6 %
Urine	1.17 ± 0.66 μg/mL	0.4%
Liver	0.28 ± 0.09 μg/g	0.1%

^a^Dose administered: Δ^**9**^THC dose administered intranasally was 268 μg/g per mouse in 126 μL solution

**Fig. 5:**
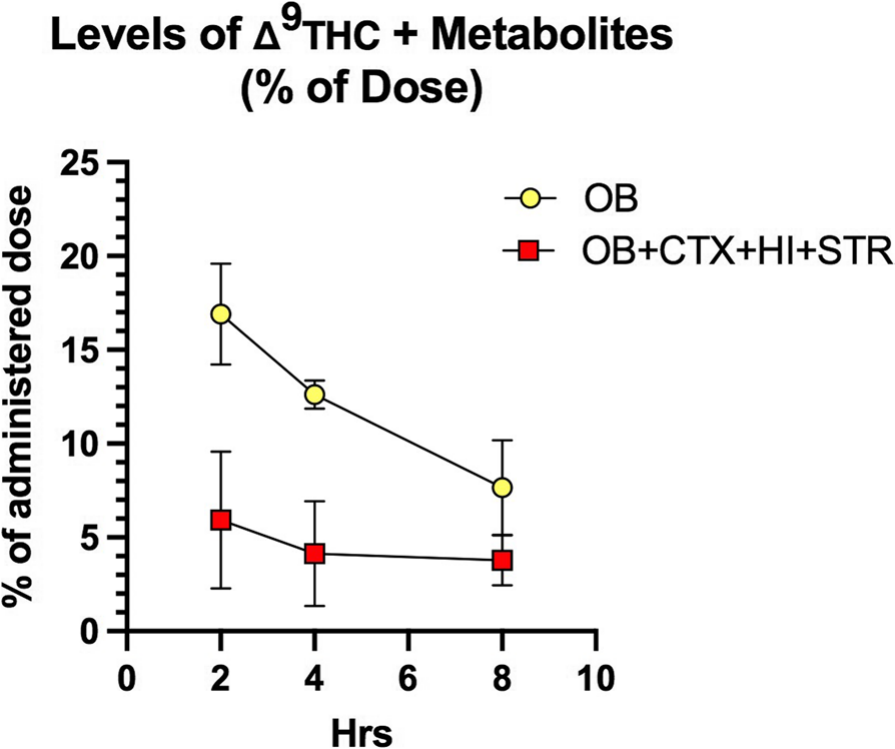
Percent of administered dose that accumulates in the brain tissues over time. Ratio (expressed as %) of mean OB concentrations to administered dose is shown in yellow circles. Percent of dose accumulated in all 4 brain regions was determined by calculating the ratio of the mean of 4 brain region concentrations of Δ^9^THC + metabolites to the administered dose of Δ^9^THC (268 μg/g). Two-way ANOVA revealed that brain regions contributed 26.9% of total variation (*p<0.005) and time contributed only 9.5% of total variation (NS). OB, olfactory bulb; CTX, cerebral cortex; HI, hippocampus; STR, striatum

For an overall estimate of transport of the drug to the brain following intranasal administration, the mean concentration of Δ^**9**^THC plus metabolites in the four brain regions was expressed as a percentage of the intranasal dose administered (268 μg/g per mouse) and compared to the levels in the olfactory lobe at 2 h (Fig. 5). There was a time-dependent decrease in the percentage of dose administered that accumulated in the olfactory bulb. However, the levels in the mean of the 4 regions of the brain remained relatively constant from 2 to 8 h. This level of bioavailability to brain after intranasal administration is higher than reported by others (Paudel et al., 2010).

## Discussion

Natural cannabis products and single phyto-cannabinoids are usually inhaled or taken orally. The rectal route, transdermal delivery, eye drops, and aerosols have only been used in a few studies. However, the oral-mucosal route has been shown to be effective and approved by the FDA, in delivering effective doses CBD (Epidiolex ^TM^) to treat intractable seizures (Nabbout & Thiele, 2020). Clearly, the pharmacokinetics of individual phyto-cannabinoids vary as a function of route of administration. Pulmonary assimilation by human of inhaled Δ^**9**^THC causes a maximum plasma concentration within minutes, psychotropic effects start within seconds to a few minutes, reach a maximum after 15–30 min, and taper off within 2–3 h (Grotenhermen, 2003). The systemic bioavailability (extent and rate at which the drug enters the systemic circulation) of inhaled Δ^**9**^THC, ranges from 10 to 35% of administered dose (Grotenhermen, 2003). Following oral ingestion, psychotropic effects set in with a delay of 30–90 min, reach their maximum after 2–3 h, and last for about 4–12 h, depending on dose and specific effect (Grotenhermen, 2003). Bioavailability Δ^**9**^THC after ingesting sesame oil or cookie ranges from 7 to 10% (Grotenhermen, 2003).

Studies of the accumulation of Δ^**9**^THC in human brain following various routes of administration cannot be performed since it requires post-mortem analysis of brain tissue. However, transport of Δ^**9**^THC to brain following various routes of administration has been explored in animal models. A recent study in rats reported mean brain levels of Δ^**9**^THC (75 ng/g), peaking at 90 min following intraperitoneal (i.p.) injections of Δ^**9**^THC (2.5 μg/g) (Baglot et al., 2021). This concentration represented 3% of the administered i.p. dose. In the present report, intranasal administration of Δ^**9**^THC resulted in peak brain concentrations at 2 h of 15.9 ng/mg, (5.9% of the administered dose). Although transport of THC to the brain following i.n. instillation in mice was almost twice that following i.p. injections in rats, the brain concentrations of THC metabolites were lower following i.n. instilation. The rat study reported higher peak concentrations of both THC-OH, (mean= 75 ng/g) and TH-COOH (12 ng/g) at 90 min, compared to a mean THC-OH of 0.5 ng/mg and mean THC-COOH of 1.67 ng/g at 2 h following i.n. route in mice. Of course, this comparison of transport of THC to the brain following i.p. vs i.n. is limited because different doses and species were utilized.

Although at this time there are no reported studies of the transport of Δ^**9**^THC from nose to brain, CBD transport into the brain following intranasal administration has been studied in rats (Paudel et al., 2010). However, in that study systemic bioavailability was determined by measuring delivery from intranasal epithelium to plasma and not to the brain. Intranasal delivery of CBD in that study used formulations that included “brain permeation enhancers” such as polyethylene glycol (PEG) in ethanol solvent (ES) and several others. The nasal absorption of CBD from all formulations was rapid, with detection at 0.5 min after intranasal administration of all the formulations, indicating rapid absorption through the nasal epithelium. Bio-availability, or uptake of CBD in venous blood, expressed as a percentage of dose administered intranasally, ranged from 0.34% to 1.4%, and this was achieved with different nasal CBD formulations. The rank order of the bioavailabilities of the various CBD formulations correlated with their lipophilicities. Interestingly, the bioavailability decreased with increasing lipophilicity of the compound. This emphasizes the need of a more polar formulation of CBD to increase the nasal permeation in the presence of epithelial mucus and other polar secretions. Unfortunately, measures of brain bio-availability following intranasal administration was not determined in the Paudel study (Paudel et al., 2010).

Other researchers have studied lipid nanocapsules (LNCs) decorated with CBD for their brain-targeting ability both in vitro and in vivo. Noticeably, the biodistribution experiments in mice consistently demonstrated that the highest brain-targeting ability was achieved with the smallest-sized CBD-decorated LNCs (Aparicio-Blanco et al., 2019). Uptake in the brain was expressed as a percentage of the intravenous injected dose and was never greater than 0.3%, even when enhancers of blood-brain-barrier penetration (CBD-decorated LNC) were attested (Aparicio-Blanco et al., 2019),

Δ^**9**^THC is readily hydroxylated to 11-OH-THC as it penetrates the blood-brain barrier, indicating that exclusive measurement of Δ^**9**^THC will never give an accurate quantitation of the ability of any formulation to enter the brain tissue itself. Furthermore, once the G-protein is activated by Δ^**9**^THC interaction with the CB1 receptor and the resulting ion flow is increased, there is no need for the preservation of the active metabolite. Oxidation of 11-OH-THC to inactive THC-COOH readily occurs after 11-OH-THC interacts with CB1R and CB2R (Sharma et al., 2012). Therefore, even the combined measurement of Δ^**9**^THC and 11-OH-THC will not give an accurate quantitation of the ability of any formulation to enter the system. While the process of G-protein activation is relatively slower than the hydroxylation of Δ^**9**^THC, the process occurs quickly, Tmax = 6 h (Calapai et al., 2020). After inactivation, the THC-COOH metabolite is excreted in urine as a glucuronic acid conjugate (Sharma et al., 2012). Once the THC-COOH is excreted, it would never be possible to detect that it was ever in the system at all, as it has no permanent effect on the system.

Once in the brain, Δ^**9**^THC can be stored in the highly lipid components of the central nervous system and results in a slower release into the brain tissue over time. This can lead to complications for quantitation of the concentration of Δ^**9**^THC in the brain as the concentration present can be impacted by Δ^**9**^THC’s complex pharmacokinetics. There is no effective quantitation method that takes all of these factors into account when exclusively measuring the concentration of Δ^**9**^THC present in the system.

Results presented here demonstrate that intranasal administration of nanoformulations of Δ^**9**^THC is a viable option for drug administration. The next iteration will be to develop a more hydrophilic nano-formulation that can enhance direct transport to the brain via the olfactory and trigeminal nerves or the perineural space and minimize immediate absorption by the capillary network of the nasal epithelium.

## Supplementary Information

**Additional file 1: Supplementary material**.
