# Supplementary material for: Measurement of Δ9THC and metabolites in the brain and peripheral tissues after intranasal instillation of a nanoformulation
Source: J Cannabis Res. 2023 Feb 7;5:3. doi: 10.1186/s42238-022-00171-8 (PMC9903512; doi:10.1186/s42238-022-00171-8)
Supplement: Supplementary file 1 — Additional file 1: Supplementary material. [file 42238_2022_171_MOESM1_ESM.pdf]

Quantitation of THC and Metabolites in Brain after Intranasal Instillation of Nanoformulations

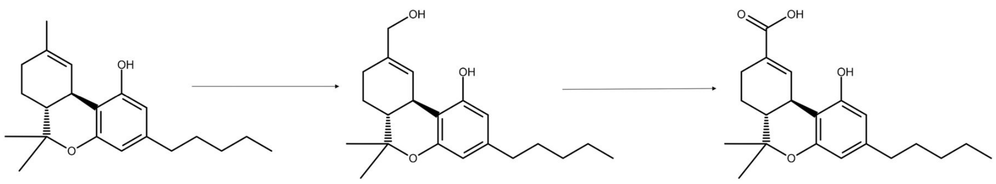

THC 11-OH-THC COOH-THC

METABOLISM AND QUANTITATION OF THC

THC is highly lipophilic, unless it is formulated into a more hydrophilic nanoparticle. Any THC released from the nanoparticle within the nasal passage will be absorbed into the rich capillary network of the nasal epithelium. Δ-9-THC is readily hydroxylated to 11-OH-THC as it penetrates the blood-brain-barrier, indicating that exclusive measurement of Δ-9-THC will never give an accurate quantitation of the ability of any formulation to enter the brain tissue itself. Furthermore, once the G-protein is activated by THC interaction with the CB1 receptor, and resulting ion flow is increased, there is no need for the preservation of the active metabolite. Oxidation of 11-OH-THC to inactive THCCOOH readily occurs after 11-OH-THC interacts with CB1R and CB2R[5]. Therefore, even the combined measurement of Δ-9-THC and 11-OH-THC will not give an accurate quantitation of the ability of any formulation to enter the system. While the process of G-protein activation is relatively slower than the hydroxylation of Δ-9-THC, the process occurs quickly, Tmax = 6 hours[2]. After inactivation, the THCCOOH metabolite is excreted in urine as a glucuronic acid conjugate[5]. Once the THCCOOH is excreted, it would never be possible to detect that it was ever in the system at all, as it has no permanent effect on the system. Studies indicate that neither Δ-9-THC, 11-OH-THC, or THCCOOH can be detected at 24 hours to a scientifically relevant amount[2].

Alternatively, Δ-9-THC can be absorbed and stored in the highly lipid components of the central nervous system and results in a slower release into the brain tissue over time. This can lead to complications for quantitation of the concentration of THC in brain as the concentration present can be impacted by THCs complex pharmacokinetic. There is not an effective quantitation method that takes all of these factors into account when exclusively measuring the concentration of Δ-9-THC present in the system.

TIME OF EXTRACTION

As Δ-9-THC is highly biologically active, proper extraction methods are vital to accurate quantitative measurements. Δ-9-THC was detectable as early as 15 minutes following subcutaneous administration[2]. Extractions that are too late after administration risk the ability to properly measure the amount of formulation taken up by the target cells. At a certain point, the cannabinoids will be undetectable to a relevant amount, though they may have been present in the target cells at some point. To avoid this complication, many tests of various extraction times must be performed, ranging from near-immediate extraction to 24 hours after administration. Alterations in the chemistry of the formulation, or concentration of THC in the formulation, will alter the Tmax, the best timepoint for extraction.

Additionally, analysis of the sample extracted must consider the metabolites. In studies in which THC is quantified, the metabolites are always present. In all ways of administration, the 11-OH-THC metabolite was shown to be present, highest in oral administration (not considering intranasal administration in this study)[2]. In a study of postmortem tissue, THC was found in 6 of 11 samples, and 11-OH-THC and THCCOOH were found in roughly similar concentrations in those same samples[6]. Over half of the data could be lost if the metabolites are not considered. The brain is not the only target tissue that demonstrates this point. Some experiments have found 11-OH-THC present in the liver after cannabis intake[2]. The ability of RadioImmunoAssay to detect THC and metabolites in blood samples is evidence that the metabolites are crucial to quantitation of the effectiveness of the formulation to enter the system and the ability of target cells to take up the cannabinoids[1]. Overall, it is important to fully analyze all relevant tissues for the cannabinoids and their metabolites.

RESOURCES

1. http://citeseerx.ist.psu.edu/viewdoc/download?doi=10.1.1.152.345&rep=rep1&type=pdf#page=41
2. https://pubmed.ncbi.nlm.nih.gov/32183416/
3. https://www.sciencedirect.com/science/article/abs/pii/S0024320511002645
4. https://www.acsh.org/news/2019/04/08/cbd-and-thc-only-difference-one-chemical-bond-13937
5. https://www.ncbi.nlm.nih.gov/pmc/articles/PMC3570572/
6. Saenz, S.R.; Lewis, R.J.; Angier, M.K.; Wagner, J.R. Postmortem fluid and tissue concentrations of THC, 11-OH-THC and THC-COOH. *J. Anal. Toxicol.* **2017**, *41*, 508–516.
